# Enhancement of viability, acid, and bile tolerance and accelerated stability in lyophilized *Weissella cibaria* JW15 with protective agents

**DOI:** 10.1002/fsn3.762

**Published:** 2018-08-22

**Authors:** Mina Kim, Dong‐Geon Nam, Sang‐Bum Kim, Pureum Im, Jeong‐Sook Choe, Ae‐Jin Choi

**Affiliations:** ^1^ Division of Functional Food & Nutrition Department of Agrofood Resources National Institute of Agricultural Science Rural Development Administration Jeonju Korea

**Keywords:** accelerated storage, freeze‐drying, lactic acid bacteria, probiotic, protective agent, *Weissella*

## Abstract

Dietary supplementation with lactic acid bacteria to maintain or improve intestinal health is advocated. *Weissella* spp. are present in different fermented vegetable‐based foods like *kimchi*, as well as in the normal gastrointestinal (GI) tract of humans. *Weissella cibaria* strains have been proposed as potential probiotics. Freeze‐drying is a promising treatment method for these strains for industrial applications and to increase the accessibility of their health‐promoting benefits. Moreover, probiotic strains need to be able to survive in the host GI tract, and acid and bile are both environmental stressors that can reduce strain survival. Therefore, this study evaluated the effect of the combination of protective agents on the acid and bile resistance of *W. cibaria* JW15 after freeze‐drying. A protective agent combination with a 1:1 ratio of 5 g + 5 g/100 ml w/v soy flour + yeast extract (SFY) retained nearly 100% viability after freeze‐drying and was resistant to artificial bile acids. Remarkably, skim milk + soy flour (SSF) was resistant to an acidic solution, and the viability of *W. cibaria* JW15 in artificial gastric acid was enhanced when treated with this mixture. Furthermore, SFY and SSF were found to maintain high numbers of viable cells with a low specific rate of cell death (*k*) after storage at 50°C, 60°C, and 70°C. These results support an effective probiotic formulation system with a high number of viable cells, and its protective effects can be leveraged in the development of probiotic products with health benefits.

## 1 INTRODUCTION

Food products containing probiotic microorganisms are gaining interest and demand since consumers started to realize their importance in health (Dianawati, Vijay, & Shah, 2016). Dietary supplementation with live lactic acid bacteria (LAB) is a promising strategy to improve host health by maintaining or improving intestinal health (Hemarajata & Versalovic, 2013). LAB has been promoted because antibiotic intake, digestive disorders, and abusive food habits may diminish the multiplication of LAB and reduce the number of healthful microbiota (King, Lin, & Liu, 1998). Indeed, LAB has attracted much attention among researchers who want to understand their roles as part of a healthy population of human microbiota (Reddy, Awasthi, Madhu, & Prapulla, 2009).

To date, beneficial LAB activity is mainly associated with *Lactobacillus* and *Bifidobacterium* genera (Parvez, Malik, Kang, & Kim, 2006); however, over the past 20 years, scientists have gained interest in the genus *Weissella* (Fessard & Remize, 2017). *Weissella* was proposed as a genus in 1993 from re‐classifying *Lactobacillus* and *Leuconostoc* spp. (Fessard & Remize, 2017). Thus, *Weissella* spp. are relatively recent members of the LAB family (Ahn et al., 2013). *Weissella*, as well as other LAB strains, are present in different fermented vegetable‐based foods which undergoes lactic acid fermentation during storage (Lim et al., 2017). *Weissella* bacteria are also present in the normal gastrointestinal tract of mammals, including humans (Fessard & Remize, 2017; Lim et al., 2017). Thus, it is reasonable to expect that *Weissella* cells enter into our bodies through our daily diet or supplements and are excreted from our bodies (Fessard & Remize, 2017). Recently, *W. cibaria* JW15 (JW15) was isolated form Kimchi, Korean traditional fermented food. Moreover, JW15 has exhibited better immuno‐modulatory effects than *Lactobacillus rhamnosus* GG and it has been suggested as a suitable probiotic supplement (Ahn et al., 2013).

To be of practical use and convenience to consumers, probiotic bacteria must be formulated a usable form often dehydrated through lyophilization (freeze‐drying) (Dianawati et al., 2016). A properly lyophilized bacteria strain can be stored for decades, without any high cooling expenses during storage or transport (Peiren et al., 2015). However, during such treatment, bacterial cells are exposed to freezing and drying processes that produce the stresses of low temperature and ice crystal formation, both of which can harmfully remove water from within the cell. Indeed, lyophilization might cause cell membrane damage and trigger a reduction in the viability of cells (Dianawati & Shah, 2011). In addition, to confer health benefits of the probiotic bacteria, cells must retain their viability during the critical stages of processing and storage, as well as during transit through the acidic stomach and bile‐rich intestine (Cheow & Hadinoto, 2013). Therefore, the potential applications of probiotic strains necessitate optimal protective agents in order to maintain survival during dehydration itself and provide stability (King et al., 1998). Even more, an appropriate protective should be effective in protecting the probiotic bacteria from the harsh environment of gastric acid and bile (Dianawati et al., 2016). Thus, it is worth noting that adequate protective agents have been explored for probiotic bacteria (Carvalho et al., 2003).

For instance, during freezing, damage to bacteria and pH changes can occur drastically, but calcium and phosphate or proteins themselves act as amphoteric molecules and increase the buffering capacity and survival rate (Pikal‐Cleland, RodrõÂguez‐Hornedo, Amidon, & Carpenter, 2000). The pure water solution was reported to freeze at −40°C. However, additional molecules as protective agents, amino acids in a heterogeneous water solution can act as seeds for ice nucleation (Janet, David, & Glick, 2014). In these conditions, a solution can freeze at high subzero temperatures, up to −2°C. Protein can induce freezing at higher temperatures, allowing a more energy efficient concentrating process (Janet et al., 2014). Skim milk powder was effective at preserving the viability of bacteria when subjected to a lyophilization process and storage, allowing its conservation and its incorporation into powdered food premixes as a probiotic additive (Gisela et al., 2014).

With that in mind, the present study aimed to identify efficient protective agents for JW15 against the lyophilization process as well as gastric acid and bile conditions. In addition, accelerated storage testing (AST) of lyophilized *W. cibaria* had not yet been reported, so the effects of a combination of protective agents on the viability of lyophilized cells were tested at various temperatures to assess the storage stability. AST is a widely used method which based on the Arrhenius theory for the prediction of long‐term preservation of lyophilized probiotics, estimation of shelf‐life, and quality (King et al., 1998).

## 2 MATERIALS AND METHODS

### 2.1 Culture preparation and feed solutions

JW15 cultures were provided by the Korean Agricultural Culture Collection (KACC 91811P). Cells were maintained on MRS agar (Difco, Detroit, MI) and cultivated in MRS broth (Difco) at 37°C using 1% inoculums. Bacteria were proliferated statically in flasks containing MRS broth with 1% inoculums and incubated at 37°C overnight. These cultures were then inoculated at 1% (v/v) in a second broth, which was incubated for 24 hr at 37°C. Cells were harvested in the stationary phase of growth by centrifugation at 15,241 × g for 15 min at 4°C and then suspended in sterile 0.85% sodium chloride solution (1% w/v on wet basis) and vortexed to yield a homogenous suspension. An individual protective agent at a specified concentration was added to the cell suspension (Table 1), that is, maltodextrin (M; ES ingredient, Gunsan, South Korea), skim milk (S; Seoul milk, Yangju, South Korea), sucrose (SC) and calcium carbonate (CC; Sigma, St Louis, MO), trehalose (T; Samyang, Ulsan, South Korea), gelatin (G; Edentown F&B, Pusan, South Korea), soy flour (SF; Tatua HSP349, Morrinsville, New Zealand), or yeast extract powder (YE; Lallemand Inc., Salutaguse Parmitehas AS, Estonia).

**Table 1:** List of protective agents for lyophilization of *Weissella cibaria* JW15

Protective agents	Contents (g/100 ml)	Abbreviation	References
Maltodextrin	10	M10	—
Maltodextrin	20	M20	Reddy et al. (2009)
Maltodextrin and glycerol	5, 2	MG	Yao, Wathelet, and Thonart (2009)
Maltodextrin, trehalose, glycerol, and NaCl	10, 15, 0.5, 1	MTGN	Jeong et al. (2015)
Trehalose	10	T10	Zhao and Zhang (2005)
Trehalose	20	T20	Reddy et al. (2009)
Skim milk	10	S10	—
Sucrose	10	SC10	Zhao and Zhang (2005)
Sucrose	20	SC20	Reddy et al. (2009)
Lactose	10	LT10	Zhao and Zhang (2005)
Lactose	20	LT20	Reddy et al. (2009)
Skim milk	20	S20	Reddy et al. (2009)
Soy flour	10	SF10	Gwak et al. (2014)
Soy flour	20	SF20	—
Yeast extract	10	YE10	—
Yeast extract	20	YE20	—
Skim milk and sucrose	10, 10	S10SC10	—
Skim milk and lactose	10, 10	S10LT10	—
Skim milk, glycerol, and CaCO_3_	10, 5, 0.1	S10GCC	Reddy et al. (2009)
Skim milk, sucrose, and gelatin	10, 8, 1.5	S10SCGT	Reddy et al. (2009)
Skim milk, trehalose, glycerol, and NaCl	10, 15, 0.5, 1	S10TGN	Jeong et al. (2015)
Lactose, gelatin, and glycerol	5, 1.5, 1	LT5GTG1	Reddy et al. (2009)
Skim milk	5	S5	—
Soy flour	5	SF5	—
Yeast extract	5	YE5	Zhao and Zhang (2005)
Skim milk and soy flour	5, 5	SSF	—
Skim milk and yeast extract	5, 5	SY	—
Soy flour and yeast extract	5, 5	SFY	—

### 2.2 Freeze‐drying

Cellular suspensions were maintained for 1 hr at room temperature prior to freezing (−80°C for 24 hr) in order to allow for equilibration between the cells and the compound added (Carvalho et al., 2003). Then, freeze drying was conducted at a shelf temperature of −40°C, cold trap temperature of −70°C and a chamber pressure of 20 mTorr for 4 days (Bondiro, programmable freeze dryer, Ilshin Co. Ltd., South Korea). The lyophilized samples were analyzed for viability and stored using airtight polythene containers. The initial population of lyophilized concentrated bacteria were 5.1 × 10^11^–7.5 × 10^11^ CFU/ml.

### 2.3 Cell viability

Residual viability of lyophilized cells was determined by the standard plate count method. The lyophilized powder (1 g) was rehydrated with 10 ml of sterile 0.85% sodium chloride solution. Next, 10‐fold serial dilutions were plated using the spread plate method. Colony forming units (CFU) were determined after 24‐hr incubation at 37°C. The percentage survival of the lyophilized sample was calculated according to the following equation:

%survival=100×Af/Bf

The variable Bf represents the viable cell count (Log CFU/ml) before freezing, and Af represents the viable cell count (Log CFU/ml) after freeze‐drying.

### 2.4 Artificial gastric acid and acid tolerance

MRS broth containing 1,000 unit/ml pepsin from porcine gastric mucosa (Sigma, Saint Louis) was adjusted to pH 3.0 using 1 N HCl to act as artificial gastric acid (Jeong et al., 2015). Sodium phosphate buffer solution (0.05 M) was adjusted to pH 3.0 with 0.1 N HCl and NaOH to make the acidic solution (Park, Lee, Kim, & Shin, 2002). One gram of lyophilized cells was inoculated into 3 ml of MRS broth and incubated for 2 hr at 37°C. After incubation, 10‐fold serial dilutions were made, and then, live cells were counted on MRS agar plates after 24 hr of incubation at 37°C. Tolerance was expressed as a percentage of the number of viable cells after incubation in artificial gastric acid MRS broth relative to that of the normal MRS broth and was calculated using the following equation (Lee, Choi, Lee, Lee, & Park, 2015):

$$\begin{array}{cc} \text{Tolerance}\;(\%)= & (\text{Log}\;\text{CFU}/\text{ml}\;\text{in}\;\text{artificial}\;\text{gastric}\;\text{acid}\;\text{or}\;\text{acidic}\;\text{solution})/\\ & (\text{Log}\;\text{CFU}/\text{ml}\;\text{in}\;\text{normal}\;\text{MRS}\;\text{broth})\times 100. \end{array}$$

### 2.5 Bile tolerance

Bile tolerance was carried out according to the method of Gilliland and Walker (1990). Briefly, 1 g of lyophilized cells was inoculated into 9 ml of MRS broth supplemented with 0.3% oxgall (Difco, Sparks, MD) and incubated for 24 hr at 37°C. Live cells were counted on MRS agar plates after incubation for 24 hr at 37°C. Bile tolerance was expressed as a percentage of the number of viable cells after incubation in MRS broth with oxgall compared to that of the control (without oxgall) and was calculated using the following equation (Lee et al., 2015):

$$\begin{array}{cc} \text{Bile}\;\text{tolerance}\;(\%)=\; & (\text{Log}\;\text{CFU}/\text{ml}\;\text{in}\;\text{MRS}\;\text{broth}\;\text{with}\;\text{oxgall})/\\ & (\text{Log}\;\text{CFU}/\text{ml}\;\text{in}\;\text{MRS}\;\text{broth}\;\text{without}\;\text{oxgall})\times 100. \end{array}$$

### 2.6 Accelerated storage test

The AST was executed according to the procedures described by Polydera, Stoforos, and Taoukis (2003). Dehydrated samples (0.2 g) in MRS broth (1 ml) were placed in a water bath at 50°C, 60°C, and 70°C. At 50°C, samples were removed and enumerated at 3‐hr intervals from 3 to 12 hr of exposure; at 60°C in 0.5‐hr intervals from 0.5 to 2 hr of exposure; and at 70°C after 0.05, 0.17, 0.25, and 0.33 hr of exposure. After thermal treatment at various temperatures, 10‐fold serial dilutions were produced by adding 0.85% sodium chloride solution, and the dilutions were cultivated on MRS agar. From the viable cell counts obtained before and after storage at temperatures of 50°C, 60°C, and 70°C, the thermal death rate was calculated as *N* ^*a*^/*N*_0_, and the *k* values of a first‐order reaction *k *=* *1/*t* (Log *N*_0_ − Log *N* ^*a*^) were calculated, where *N*_0_ is the initial number of cells, *N* ^*a*^ is the residual number of cells after the time indicated (*t*), and *k* is the rate constant (slope) expressed in the unit of Log_10_ number of cells per unit of time in hours (an experimental constant). The effect of temperature on the specific reaction rate was represented by the Arrhenius equation (King et al., 1998):

$$\text{Log}\;k=\text{Log}\;k_{0}-\left(E_{a}/R\right)\left(1/T\right)$$

where *T* is the absolute temperature, *R* is the gas constant, and *E*_a_ is the energy of activation.

Based on the climate of South Korea, the number of days at each temperature was as follows: 10°C (5 months), 15°C (1 month), 20°C (2 months), 25°C (2 months), and 30°C (2 months) (Park, An, Lee, & Park, 2014). Based on a lower limit standard (cell number 10^8^ CFU/g (Dianawati et al., 2016; Savedboworn et al., 2017)), shelf life was calculated as follows (Park et al., 2014):

Shelflife=[(LogA−LogB)/C]×365

where *A* is cell number of before thermal treatment, *B* is the lower limit of cell number, and *C* is the sum of each month variation (number of months at each temperature × *k* value at each temperature).

### 2.7 Statistical analysis

Experiments were carried out in triplicate to evaluate the probiotic properties. The mean and standard deviation were calculated for *n* = 3. Their levels of significance were assessed with a one‐way ANOVA followed by a Duncan's Multiple Range Test in SPSS 18.0 (Chicago, IL) with a significance level of *p *<* *0.05.

## 3 RESULTS AND DISCUSSION

### 3.1 Effects of protective agents on the viability of *W. cibaria* JW15 during the lyophilization

**Figure 1:**
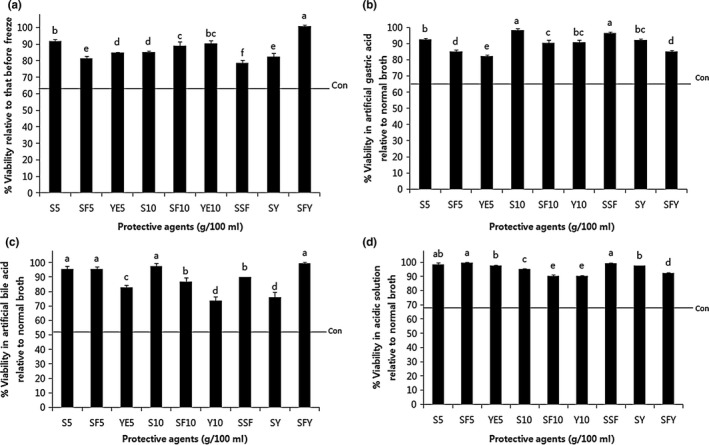
Viability of *Weissella cibaria* JW15 against (a) lyophilization, (b) artificial gastric acid, (c) artificial bile, and (d) acidic solution after lyophilization with combinations of different protective agents. Values with different lowercase letters (a–f) are significantly different by Duncan's multiple range test (*p *<* *0.05). S5, skim milk 5 g; SF5, soy flour 5 g; YE5, yeast extract 5 g; S10, skim milk 10 g; SF10, soy flour 10 g; YE10, yeast extract 10 g; SSF, skim milk 5 g + soy flour 5 g (10 g total); SY, skim milk 5 g + yeast extract 5 g (10 g total); SFY, soy flour 5 g + yeast extract 5 g (10 g total); Con, control, without protective agent (a, 63%; b, 65%; c, 52%; d, 69%)

The use of LAB in the food industry depends on the concentration and preservation technologies employed, which are required to guarantee long‐term delivery of microbe viability and functional activity (Carvalho et al., 2003). To our knowledge, these characteristics of JW15 have not been previously described. Therefore, as a first step in the investigation of the effects of protective agents against lyophilization injury to JW15, 22 additives were tested (Table 2). After lyophilization, an obvious decrease in cell viability occurred in the control group (without protective agent). On the other hand, YE10 showed significantly higher levels of cell viability after lyophilization, and SF10 was comparable. Maximum viability was obtained with YE10 (91%), followed by SF10 (90%) and S10 (85%). A recent study reported that skim milk, yeast extract, and soy powder were used as protective agents for *W. cibaria* SW1‐1 during the lyophilization (Gwak et al., 2014). The best results were obtained with 10% soy powder—approximately 90% cell viability was observed. Interestingly, a high dose of soy powder did not show a positive effect compared with a low dose (Gwak et al., 2014). Consistent with this, increasing the concentration of protective agents from 10 to 20 g did not cause a proportional enhancement in the survival rate of JW15 (Table 2). This result led us to apply a lower dose (5 g) and a mixture using several selected effective protective agents (i.e., S, SF, and YE). As shown in Figure 1A, the mixture of soy flour and yeast extract powder (SFY, 1:1 ratio, total 10 g/100 ml) was the most effective protective agent in JW15 cells, followed by S5, YE10, and SF10.

**Table 2:** Comparative effects of different protective agents on *Weissella cibaria* JW15 viability after lyophilization

Protective agents (g/100 ml)	Viability (%)
YE10	91.13 ± 1.12^a^
SF10	89.99 ± 0.52^ab^
S10GCC	87.29 ± 1.12^bc^
YE20	86.98 ± 0.21^bc^
SF20	84.77 ± 1.10^cd^
S10	84.59 ± 0.89^cd^
S10LT10	82.03 ± 1.29^de^
S10SC10	81.86 ± 1.87^de^
S20	81.37 ± 0.60^de^
M10	80.24 ± 0.37^ef^
S10TGN	78.94 ± 1.78^efg^
M20	77.04 ± 0.34^fgh^
LT10	76.41 ± 2.44^gh^
LT5GTG1	74.10 ± 1.75^hi^
T10	73.04 ± 0.78^i^
LT20	71.62 ± 1.54^i^
T20	71.10 ± 0.57^ij^
MTGN	68.32 ± 2.16^jk^
MG	67.29 ± 4.87^k^
S10SCGT	65.14 ± 4.75^k^
SC10	61.26 ± 2.60^l^
SC20	59.76 ± 0.00^l^
Control	61.55 ± 2.39^l^

*Notes*

Values with different lowercase letters (a–l) are significantly different by Duncan's multiple range test (*p *<* *0.05).

YE10, yeast extract 10 g; SF10, soy flour 10 g; S10GCC, skim milk 10 g + glycerol 5 g + CaCO_3_ 0.1 g; YE20, yeast extract 20 g; SF20, soy flour 20 g; S10, skim milk 10 g; S10LT10, skim milk 10 g + lactose 10 g; S10SC10, skim milk 10 g + sucrose 10 g; S20, skim milk 20 g; M10, maltodextrin 10 g; S10TGN, skim milk 10 g + trehalose 15 g + glycerol 0.5 g + NaCl 1 g; M20, maltodextrin 20 g; LT10, lactose 10 g; LT5GTG1, lactose 5 g + gelatin 1.5 g + glycerol 1 g; T10, trehalose 10 g; LT20, lactose 20 g; T20, trehalose 20 g; MTGN, maltodextrin 10 g + trehalose 15 g + glycerol 0.5 g + NaCl 1 g; MG, maltodextrin 5 g + glycerol 2 g; S10SCGT, skim milk 10 g + sucrose 8 g + gelatin 1.5 g; SC10, sucrose 10 g; SC20, sucrose 20 g; Control, without protective agent.

According to the results of Zhao and Zhang (2005), yeast extract was the best protective agent for *Lactobacillus brevis* and *Oenococcus oeni*, with a cell viability of 67.8 and 53.6%, respectively. The soy flour used in the current study contained 9.7% total nitrogen, amino nitrogen (1.9%), and minerals (Ca, 62 mg/100 g; Cl, 99 mg/100 g; P, 310 mg/100 g; K, 3 g/100 g; and Na, 1 g/100 g). This protection by soy flour and yeast extract within the amino acids category was thought to be the result of a reaction between the carboxyl groups of the bacterial proteins and the amino group of the protective agent, stabilizing the proteins structure (Moriche, 1970). Particularly, yeast extract contains many amino acids, mainly glutamate (Zhao & Zhang, 2005). Glutamate usually in combination with other compounds such as milk was effective in cryoprotection (Hubalek, 2003). Together, the results indicate that soy flour, yeast extract, and skim milk can be useful protective agents for JW15.

### 3.2 *Weissella cibaria* JW15 tolerance in acidic and bile environments

In a previous study, survival of cultured JW15 was evaluated at pH 3.0 and in bile conditions (Ahn et al., 2013). JW15 could survive at pH 3.0 for 2 hr. It was also showed tolerance to 0.3% oxgall bile salt. Here, acid and bile tolerance of lyophilized JW15 with protective agents was demonstrated for verification of the possibility of a probiotic application (Figure 1B–D).

Typically, the pH of gastric acid in the stomach is in the range of 3.0–3.5 (Dianawati et al., 2016). Patel, Lindström, Patel, Prajapati, and Holst (2012) reported that *W. cibaria* 142 showed 131% survival after 2.5 hr at 37°C in MRS broth adjusted to pH 3, which demonstrated that the strain not only survives, but proliferates in acidic conditions. However, *W. cibaria* 92 was sensitive to acidic conditions (16% survival). In the current study, after culturing lyophilized JW15 in the artificial gastric acid, the survival rate was more than 90% in the presence of S10, SF10, YE10, SSF, and SY compared with normal MRS broth (Figure 1B). Specifically, SSF showed the highest (97%) gastric acid tolerance. Likewise, lyophilized JW15 with a protective agent was generally capable of surviving well (i.e., more than 90%) in an acidic solution (Figure 1D). In particular, S5 and SF5—including SSF—almost reached 100% viability.

Several factors such as type of proteins and concentration are important for the growth and maintenance of viability of probiotics in food matrices. The product formulation plays an important role in the efficacy of probiotics (Vinderola & Reinheimer, 2003). Skim milk powder prevents cellular injury by stabilizing the cell membrane constituents and creating a porous structure (Abadias, Benabarre, Teixido, Usall, & Vinas, 2001). Casein improved viability of probiotics after lyophilization and during storage (Heidebach, Petra, & Kulozik, 2010). In addition, skim milk contains proteins that provide a protective coating for the cells and has excellent buffering capacity, which provides good shielding for LAB during transit in the acidic environment of the stomach (Livney, 2010). Skim milk has been used alone or with other compounds as a support material in combination (Abadias et al., 2001).

Following exposure to an acidic environment in the stomach, bacteria are exposed to the bile environment in the small intestine. Bile functions as a biological detergent emulsifying lipids when chyme is released into the duodenum (Patel et al., 2012). In addition, bile possesses strong antimicrobial activity, because it is able to disorganize the structure of the cell membrane and trigger DNA damage (Lorena, Abelardo, & Sánchez, 2013). In this sense, bile tolerance is one of the most crucial properties for probiotic bacteria, as it determines its ability to survive in the small intestine, and consequently its capacity to play a functional role. Moreover, bile‐adapted bacteria usually confer cross resistance to other stress factors (Lorena et al., 2013). The actual physiological concentration of human bile in the duodenum is in the range of 0.3%–0.5% (Vinderola & Reinheimer, 2003). Results from a previous study reported that *W. confuse* AI10 was the most resistant strain to bile salts, with 72% survival after 24 hr at 37°C, and *W. cibaria* 92 and 142 showed survival rates of 33% and 54%, respectively (Patel et al., 2012). A recent study reported that *W. cibaria* WD2 exhibited tolerance to a stimulated gastric environment with intestinal transit, tolerated acid, and bile salts and was safe for human consumption (Ojekunle, Banwo, & Sanni, 2017).

Lyophilized JW15 with protective agents was able to survive in the presence of 0.3% bile (oxgall), as shown in Figure 1C. Dehydrated JW15 with SFY appeared the most stable in bile environments. These data also indicate that the viability of S10 and SF5 groups was 98% and 96%, respectively. Similarly, Shimakawa, Matsubara, Yuki, Ikeda, and Ishikawa (2003) observed that the inhibition of *Bifidobacterium* by bile was partially alleviated by addition of soy protein, which has been shown to bind and aggregate bile acids. In case of *Lactobacillus*, it was rapidly inactivated by bile in the small intestinal model; however, addition of meat exerted a protective effect and resulted in increased delivery of cells to the intestinal compartment (Ganzle, Hertel, van der Vossen, & Hammes, 1999). In another study, *Lactobacillus plantarum* with soy lecithin improved the viability at a concentration of 0.3% bile (Hu et al., 2015).

Survival under acidic and bile conditions such as in the human gastrointestinal tract is an important consideration for LAB, and resistance to these conditions is crucial criterion in selection of a specific probiotic strain (Termont et al., 2006). The observations in this study provide support regarding resistance of JW15 with SSF or SFY to bile and acidic conditions. In this respect, these protective agents might be provisionally accepted for probiotic protection in supplement applications.

### 3.3 Prediction of the storage stability of lyophilized *W. cibaria* JW15 using an accelerated storage test

**Figure 2:**
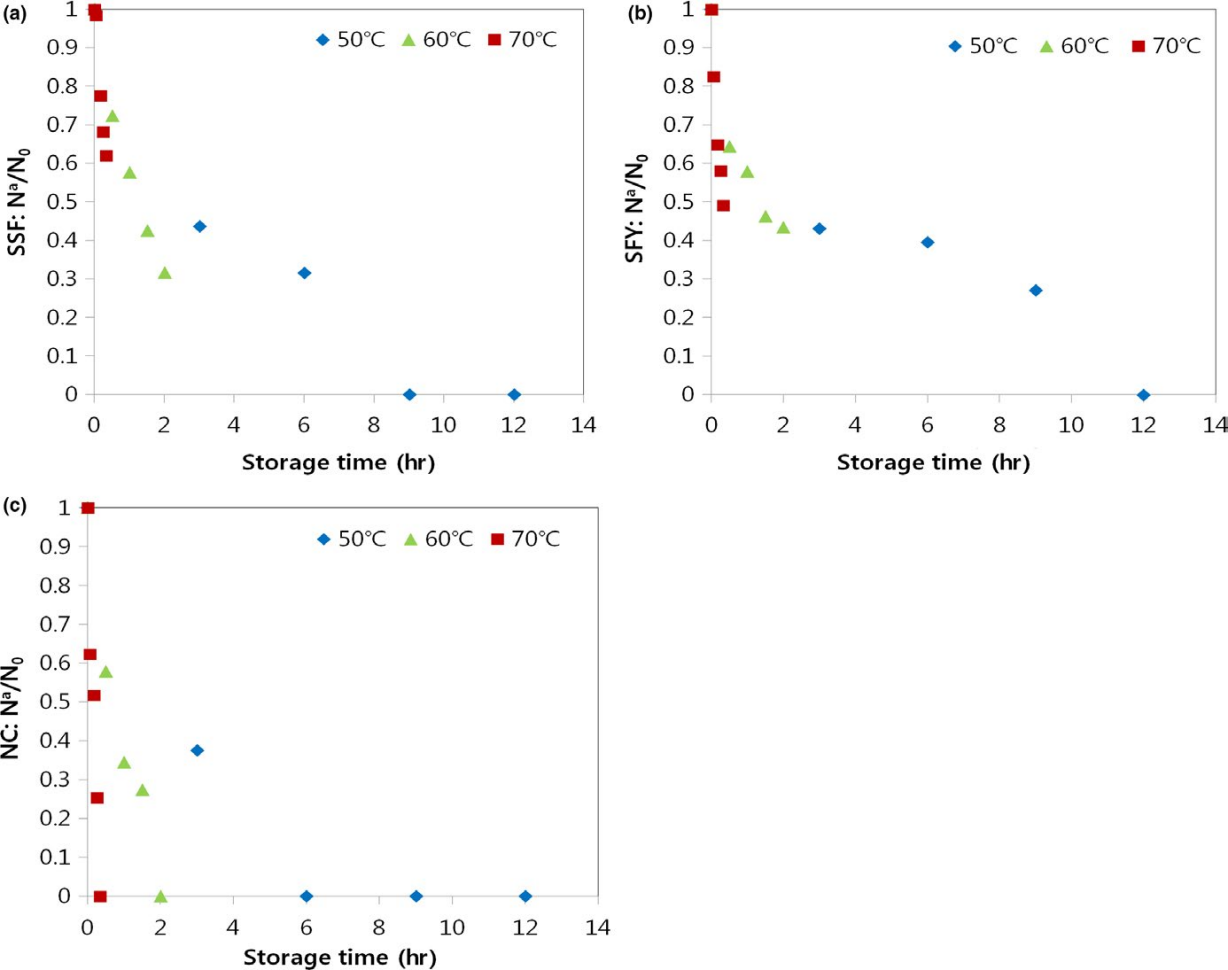
Effect of storage time at different temperatures on the thermal death of lyophilized *Weissella cibaria* JW15 with (a) SSF, (b) SFY, or (c) NC. *N*_0_, initial number of viable cells; *N* ^*a*^, number of viable cells after the time indicated. SSF, skim milk 5 g + soy flour 5 g; SFY, soy flour 5 g + yeast extract 5 g; NC, no protective agent added control

The stability of lyophilized bacteria largely depends on the storage temperature. AST are performed by incubating the samples at temperatures higher than the usual storage temperature (50°C, 60°C, and 70°C). The number of decimal reductions in lyophilized JW15 with or without protective agents (i.e., the combination of skim milk, soy flour, and yeast extract powder) exposed to three different temperatures is presented in Figure 2. Lyophilization without protective agents (nontreatment control, NC) produced the most degradation in all samples tested (Figure 2c), and thermal death at 70°C was the fastest among the three temperatures applied. Similarly, another study demonstrated that *Lactobacillus acidophilus* with protective agents degraded more significantly at 70°C compared to low temperature (King et al., 1998). Meanwhile, the thermal death of cells was lower with SSF and SFY compared to NC during the same period (Figure 2a,b). This result was in agreement with Savedboworn et al. (2017), who demonstrated that, even though a greater thermal reduction was produced at a higher temperature, skim milk, and protein protective agents effectively decreased thermal death. After exposure to 50°C, very few cells of NC and SSF were detected at 6 and 9 hr, respectively (Figure 2a,c). On the other hand, cells at 50°C were detected in the SFY group until 12 hr (Figure 2b). This indicates that the application of SFY improved survival during storage.

Then, data obtained from Figure 2 are used for performing extrapolation studies and prediction of the real‐time behavior. The *k* values are shown in Table 3. Those values are significantly different and ranged from 1.415 to 35.477 hr^−1^, the lowest value being for the lyophilized bacteria with SFY at 50°C and the highest value for the lyophilized bacteria without protective agent at 70°C. The application of protective agents was found to decrease the *k* values compared to lyophilization without protective agent.

**Table 3:** The *k* (hr^−1^) values for the thermal reductions of lyophilization with or without different protective agents

Temperature (°C)	Lyophilization without protective agents	Lyophilization with SSF	Lyophilization with SFY
50	2.45 ± 0.05	1.78 ± 0.48	1.42 ± 0.57
60	7.46 ± 1.75	4.58 ± 0.89	5.79 ± 2.21
70	35.48 ± 1.48	15.71 ± 1.16	27.84 ± 16.58

*Note*

SSF, skim milk 5 g + soy flour 5 g; SFY, soy flour 5 g + yeast extract 5 g.

**Figure 3:**
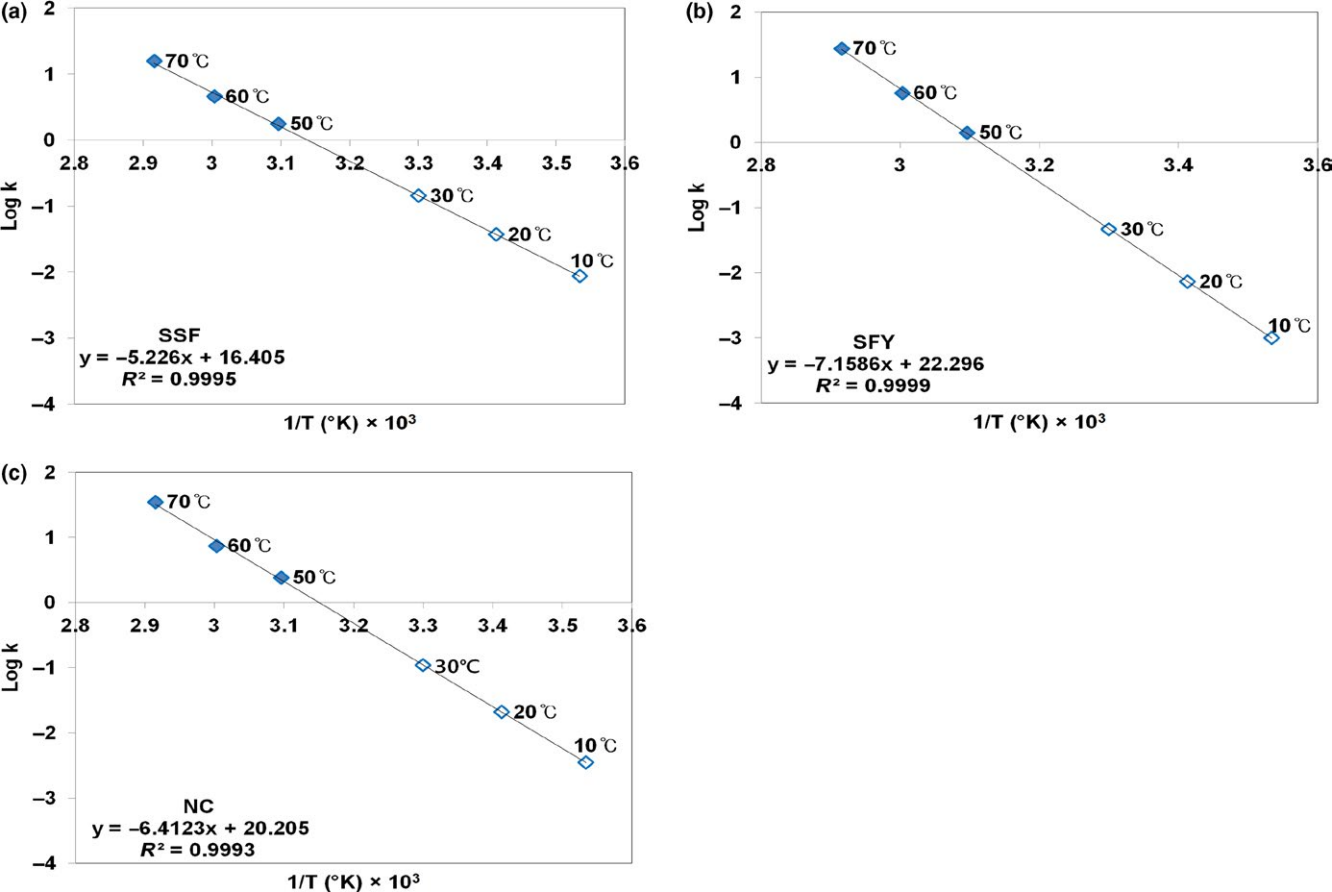
Arrhenius plots of the specific rate of cell death (*k*) of lyophilized *Weissella cibaria* JW15 with (a) SSF, (b) SFY, or (c) NC at various temperatures. SSF, skim milk 5 g + soy flour 5 g; SFY, soy flour 5 g + yeast extract 5 g; NC, no protective agent added control; ◆, experimental; ◇, predicted

Subsequently, the AST method at 50°C, 60°C, and 70°C was further used for predicting the stability at 10°C, 20°C, and 30°C of lyophilized JW15 with or without protective agents in order to compare them. The Arrhenius equation is the most common and generally valid model of the temperature dependence of the deterioration rate. By means of the Arrhenius relationship, the stability of the lyophilized bacteria was predicted using the AST method. From the results of Table 3, when the logarithms of the determined *k* values were plotted against the reciprocals of their absolute temperatures, straight lines were obtained in Figure 3. From the slopes of those three lines, it was found that the effect of storage temperature was most significant on the lyophilized JW15 with SFY (Figure 3b). Therefore, with regard to those three tests, the most effect was achieved with SFY, and the worst was with NC.

For commercial probiotic products, it is very important to keep the stability of live microorganisms throughout the shelf life of the products. The shelf life of lyophilized JW15 with or without protective agents was estimated. Probiotic foods with health claims should contain at least 10^6^ CFU/ml of the bacteria on expiry because 10^8^–10^9^ cells is the minimum therapeutic dose per day (Shimakawa et al., 2003). Probiotic supplement products are often refrigerated to keep the bacteria alive with a minimum amount of 10^7^ CFU/g (Dianawati et al., 2016). Commercial probiotics as food supplements require bacteria at 10^7^ CFU/g before being consumed. Besides, a previous study recommended dose of probiotic products to be over 8 log CFU/ml (Savedboworn et al., 2017). Therefore, 10^8^ CFU/g was decided to be an appropriate lower limit. As a result, based on the climate in South Korea and lower limit of cell amount, its predicted shelf life at ambient condition was calculated using the result of Figure 3, Arrhenius plots. The application of SFY was found to preserve the cells for a longer period compared to that lyophilization without protective agents (SFY, 441 days; NC, 157 days). The addition of SFY as a protective agent could effectively increase the storage stability of dehydrated JW15 over 1 year. This result found the efficient protective agents which have a great capability to stabilize JW15 during lyophilization and storage. The ability of protective agents, protein, and protein supplement (i.e.*,* soy flour and yeast extract) to enhance the stability of the JW15 during subsequent storage was decided.

## 4 CONCLUSION

The addition of soy flour and yeast extract powder (SFY, 5 g + 5 g/100 ml w/v) as a protective agent during the lyophilization can maintain the viability of JW15, ensuring the proper administration of probiotics to their destinations. Furthermore, SFY was evaluated for its effects on storage stability of the lyophilized powder through an accelerated storage test. We found that this protective agent is an effective method for protecting the health benefits of probiotic products and is desirable for industrial development.

## ETHICAL STATMENT

Neither animal nor human testing was not involved in this study.

## CONFLICT OF INTEREST

The authors declare no conflict of interest.
